# Effects of Low-Level Er:YAG Laser Irradiation on Proliferation and Calcification of Primary Osteoblast-Like Cells Isolated From Rat Calvaria

**DOI:** 10.3389/fcell.2020.00459

**Published:** 2020-06-23

**Authors:** Hiromi Niimi, Yujin Ohsugi, Sayaka Katagiri, Kazuki Watanabe, Masahiro Hatasa, Tsuyoshi Shimohira, Yosuke Tsuchiya, Shogo Maekawa, Tomomitsu Hirota, Hiroshi Kadokura, Satoshi Yokose, Takanori Iwata, Akira Aoki

**Affiliations:** ^1^Department of Periodontology, Graduate School of Medical and Dental Sciences, Tokyo Medical and Dental University, Tokyo, Japan; ^2^Division of Molecular Genetics, Research Center for Medical Sciences, The Jikei University School of Medicine, Tokyo, Japan; ^3^Division of Endodontic and Operative Dentistry, Department of Restorative and Biomaterials Sciences, School of Dentistry, Meikai University, Saitama, Japan

**Keywords:** Er:YAG laser, osteoblast-like cell, microarray, proliferation, calcification, gene expression

## Abstract

Several reports have shown that the photo-bio-modulation of cells by various lasers has favorable biological effects. However, the effects of low-level Er:YAG laser irradiation on osteoblasts remain unclear. The purpose of this study was to evaluate the effects of low-level Er:YAG laser irradiation on proliferation and osteogenic differentiation of primary osteoblast-like cells isolated from the calvariae of 3–5-day-old Wistar rats. Cells were irradiated by Er:YAG laser at energy fluences of 2.2, 3.3, and 4.3 J/cm^2^, respectively. After irradiation, cell surface temperatures were measured and cell proliferation was evaluated by flow cytometry and CCK-8. Calcification was evaluated by measuring areas of Alizarin red S staining after 7, 14, and 21 days culture in osteoinductive medium. Gene expression in non-irradiated and laser-irradiated cells was evaluated by qPCR at 3, 6, and 12 h, as well as 1, 3, 7, and 14 days after irradiation. Microarray analysis was performed to comprehensively evaluate the gene expression of non-irradiated and irradiated cells at 3.3 J/cm^2^ at 6 h after irradiation. No pronounced increase of cell surface temperature was induced by irradiation. Irradiation did not affect osteoblast-like cell proliferation. Osteoblast-like cell calcification was significantly increased 7 days after Er:YAG laser irradiation at 3.3 J/cm^2^. *Bglap* expression was significantly increased in cells irradiated at 3.3 J/cm^2^ 6 h post-irradiation. Microarray analysis showed that irradiation at 3.3 J/cm^2^ caused an upregulation of inflammation-related genes and downregulation of *Wisp2*. Gene set enrichment analysis also clarified enrichment of inflammation-related and Notch signaling gene sets. In conclusion, low-level Er:YAG laser irradiation at 3.3 J/cm^2^ enhanced calcification of primary osteoblast-like cells via enhanced *Bglap* expression and enriched Notch signaling.

## Introduction

Photo-bio-modulation of low-level laser therapy (LLLT) has beneficial effects on multiple cell types (Huang et al., 2009; AlGhamdi et al., 2012); consequently, it has been used in medical and dental clinical practice to enhance tissue healing (Stein et al., 2008). Several previous reports have shown that LLLT promotes anti-inflammation (Avci et al., 2013), pain relief (Jang and Lee, 2012; Carroll et al., 2014), wound healing (Woodruff et al., 2004; Noda et al., 2016), and tissue regeneration (Pourzarandian et al., 2004; Rizzi et al., 2006; Avci et al., 2013). In particular, because enhanced bone regeneration is required in some fields of medicine and dentistry including periodontal therapy (Aoki et al., 2015), the number of reports on the application of LLLT to regenerate bone tissue has been increasing (Pourzarandian et al., 2004; Noda et al., 2016). It is known that osteoblasts play essential roles in bone formation and remodeling (Hadjidakis and Androulakis, 2006); therefore, biostimulation by LLLT on osteoblasts needs to be investigated.

Some types of lasers have been reported to have biostimulation effects on osteoblasts; for example, we previously observed enhanced differentiation and calcification of MC3T3-E1 by blue laser irradiation (Mikami et al., 2018) and others have reported that human osteoblast proliferation and differentiation were promoted *in vitro* following irradiation by He-Ne (Stein et al., 2005) or Nd:YAG lasers (Arisu et al., 2006). Irradiation by Ga-Al-As diode laser was also reported to promote proliferation, differentiation, and bone-nodule formation of primary osteoblast-like cells isolated from rat calvariae (Ozawa et al., 1998; Ueda and Shimizu, 2003; Shimizu et al., 2007). In addition, Grassi et al. (2011) showed that low-level laser treatment enhanced cell calcification, but not proliferation in osteoblast-like cells.

Regarding Er:YAG laser, which is most effectively used in periodontal regenerative therapy (Aoki et al., 2015), we previously reported that low-level irradiation increased proliferation of MC3T3-E1 (Aleksic et al., 2010). However, compared to other types of lasers, there are still relatively few reports on the effects of low-level Er:YAG laser irradiation on the proliferation of osteoblasts. Furthermore, calcification of osteoblasts irradiated by Er:YAG laser has never been evaluated, and there are no reports offering a comprehensive analysis of gene expression in irradiated osteoblasts. Available evidence on the biostimulatory effects of low-level Er:YAG laser irradiation on osteoblasts remains limited. Therefore, the purpose of this study was to evaluate the effects of low-level Er:YAG laser irradiation on proliferation and osteogenic differentiation of primary osteoblast-like cells. In addition, comprehensive gene expression analysis was conducted to clarify the influence of laser irradiation on osteoblast-like cells.

## Materials and Methods

### Cell Isolation and Culture

Osteoblast-like cells were isolated from the calvariae of 3–5-day-old Wistar rats (Sankyo Labo Service Corporation, Tokyo, Japan) as described previously (Yokose et al., 1996; Gu et al., 2006). Calvariae without periosteums were aseptically dissected and processed by serial enzymatic digestion. Briefly, the calvariae were cut into pieces using scissors, which were suspended in 3 mL enzyme mixture and incubated in a water bath shaker at 37°C for 20 min. After the incubation, the supernatant containing released cells were collected in a new tube and mixed with an equal volume of growth medium. The growth medium was alpha minimal essential medium (α-MEM; Wako, Osaka, Japan), supplemented with 10% fetal bovine serum (FBS; Gibco, Carlsbad, CA, United States) and 1% antibiotic-antimycotic mixture (Invitrogen, Carlsbad, CA, United States). This enzymatic digestion was repeated four times; the cells isolated from the last three fractions, which are abundant in osteoblast-like cells (Gu et al., 2006), were used in all experiments. All protocols for animal use and euthanasia were approved by the Animal Care Committee of the Experimental Animal Center at Tokyo Medical and Dental University (A2019-098C3).

Cells were precultured in 10-cm culture dishes in growth medium. When the cells reached 80% confluency, they were seeded in 35-mm dishes for cell proliferation assay, calcification assay, and evaluation of gene expression. All cultures were maintained in a humidified atmosphere of 95% air and 5% CO_2_ at 37°C. The medium was changed every 3 days.

### Laser Irradiation

An Er:YAG laser apparatus (DELight; HOYA ConBio, Fremont, CA, United States) emitting at a wavelength of 2.94 μm was employed in this study. Laser irradiation was performed perpendicularly to the bottom of the culture dish at a distance of 25 cm, with the handpiece fixed using a stand as described previously (Aleksic et al., 2010). To completely irradiate the 35-mm dish, neither cover sleeve nor contact tip was mounted with the handpiece. The medium was removed immediately before irradiation and all irradiations were performed in the absence of culture medium. The output energy settings were 35, 55, 70 mJ/pulse and 20 Hz on the panel, with an irradiation time of 60 s. The actual energy levels at the dish surface were 17.6, 26.4, 34.5 mJ/pulse, and the actual energy densities were 1.8, 2.7, 3.6 mJ/pulse/cm^2^, resulting in total energy densities of 2.2, 3.3, 4.3 J/cm^2^, respectively. Immediately after irradiation, the appropriate medium was added to the dish, according to the subsequent experiments.

### Surface Temperature Measurement

Cell surface temperatures were measured before, during, and after laser treatment using a non-contact infrared laser thermometer (wavelength 630–670 nm, output <1 mW; MiniTemp, RayTek Corporation, Santa Cruz, CA, United States) on monolayer MC3T3-E1 cells at room temperature (22–23°C).

### Evaluation of Cell Proliferation

Osteoblast-like cells seeded at 1 × 10^3^ per dish were cultured for 48 h with growth medium, then serum-starved by replacing with starvation medium (α-MEM containing 0.5% FBS and 1% antibiotic-antimycotic mixture) for an additional 24 h to induce cell-cycle synchronization. Prior to laser irradiation, the cells were labeled with 0.5 μL of 5 μM CellTrace Violet (Thermo Fisher Scientific, Waltham, MA, United States) according to the manufacturer’s protocol. At 24 h following irradiation, the cells were analyzed using an Attune NxT flow cytometer (Thermo Fisher Scientific). As an initial generation control, cells were labeled with CellTrace Violet immediately before cytometry. Non-labeled cells were used as a negative control.

We also evaluated cell proliferation using the Cell Counting Kit-8 (CCK-8, Dojindo, Kumamoto, Japan) at 1, 2, and 3 days after laser irradiation as described previously (Aleksic et al., 2010; Kong et al., 2018). Laser irradiation was performed in the absence of medium after a 24 h-starvation. Immediately after irradiation, dishes were refilled with growth medium. CCK-8 was applied to non-irradiated control and irradiated cells at 1, 2, and 3 days after laser irradiation. A 1:10 mixture of kit solution and α-MEM (750 μL) was added to the cultured osteoblast-like cells and the dishes were incubated at 37°C for 30 min. The optical absorbance of samples was measured using a fluorescence microplate reader at a wavelength of 450 nm (FMAX, Molecular Devices, Sunnyvale, CA, United States). Relative cell proliferation was expressed as a ratio of proliferation activity of irradiated cells to that of non-irradiated cells.

### Calcification Assay

Osteoblast-like cells were seeded at 5 × 10^4^ cells per dish. When cells reached confluency, laser irradiations were performed in the absence of the medium. Immediately after irradiation, the dishes were re-filled with osteoinduction medium [growth medium containing 50 μg/mL ascorbic acid (Wako, Osaka, Japan), 10 mM β-glycerophosphate (Sigma-Aldrich), and 10 nM dexamethasone (Sigma-Aldrich)] for osteoinduction (Ozawa et al., 1998). At 7, 14, and 21 days following irradiation, dishes were stained with 1% Alizarin red S to assess calcified matrix production. Relative areas of staining of irradiated and non-irradiated cells were measured using Photoshop CC software (Adobe Systems Inc., San Jose, CA, United States).

Quantitative evaluation of Alizarin red S staining by solubilization was performed and absorbance measured as described previously (Gregory et al., 2004). Briefly, dye from stained monolayers was extracted by 10% acetic acid and neutralized with 10% ammonium hydroxide, and absorbance of the supernatants was detected at 405 nm. Relative cell calcification is expressed as absorbance relative to that of non-irradiated cells.

### Quantitative Polymerase Chain Reaction

Osteoblast-like cells were seeded at 5 × 10^4^ per dish. When cells reached confluency, laser irradiations were performed in the absence of medium. Immediately after irradiation, the dishes were re-filled with osteoinduction medium as described above. Cells were collected for RNA extraction at 3, 6, and 12 h, as well as 1, 3, 7, and 14 days, after irradiation.

Total RNA was extracted using Trizol reagent (Invitrogen, Carlsbad, CA, United States) with NucleoSpin RNA (TaKaRa Bio, Shiga, Japan). Complementary DNA was synthesized using a PrimeScript RT Master Mix (TaKaRa Bio) according to the manufacturer’s instructions. Quantitative polymerase chain reaction (qPCR) was performed using SYBR Premix Ex Taq II (TaKaRa Bio) and Thermal Cycler Dice Real Time System II (TaKaRa Bio). The *18s* rRNA expression level was used as an internal control. The results are shown as a ratio of the gene expression level relative to the non-irradiated control samples from the same time point. PCR primers are listed in Supplementary Table S1.

### Microarray Analysis and Validation

The Agilent Low Input Quick Amp Labeling kit (Agilent Technologies, Santa Clara, CA, United States) was used to generate cRNA from an input of 200 ng total RNA for single-color (Cy3) microarray analysis. Then, cRNAs were analyzed by hybridization onto an Agilent SurePrint G3 Unrestricted Gene Expression 8 × 60 K Microarray (Agilent Technologies). Fluorescence signals were detected using the Agilent Microarray Scanner System (Agilent Technologies). Raw microarray data were extracted using Feature Extraction Software (ver. 11.0.1.1; Agilent Technologies).

### Statistical Analysis

Data are presented as means ± standard error (SE). Experiments were repeated six times unless stated otherwise. Data distributions were assessed with the Shapiro–Wilk test. Paired *t*-tests with Bonferroni corrections were performed for multiple-group comparisons in parametric data. For non-parametric data, the Steel method was applied for comparisons with control samples. Data were analyzed using JMP v.9 software (SAS Institute Inc., Cary, NC, United States). *P* < 0.05 was considered statistically significant.

Microarray data were quantile normalized and log_2_-transformed (Shippy et al., 2006) using R (ver. 3.6.0). The Limma Bioconductor package (ver. 3.40.6) (Ritchie et al., 2015) was used to identify differentially expressed genes. Gene set enrichment analysis (GSEA^1^; Subramanian et al., 2005) was carried out with hallmark gene sets (Liberzon et al., 2015).

## Results

### Surface Temperatures

**FIGURE 1:**
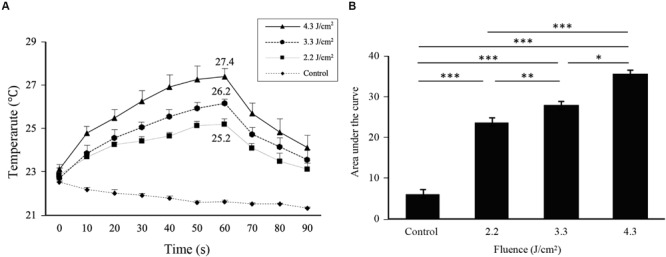
Cell surface temperatures change during and after Er:YAG laser irradiation. **(A)** Time course of changes in the cell surface temperature of MC3T3-E1 cells depending on the irradiation levels. **(B)** The area under the curve calculated from each curve in **(A)** relative to a baseline of 21°C (means ± SE, *n* = 6). *P < 0.05, **P < 0.01, and ***P < 0.001 (paired *t*-test with Bonferroni correction).

Irradiation at 2.2, 3.3, and 4.3 J/cm^2^ increased the surface temperatures of cell monolayers from 22.8 ± 0.1°C before irradiation to 25.2 ± 0.1, 26.2 ± 0.1, and 27.4 ± 0.2°C, respectively, with an average increase of 2.4, 3.4, and 4.6°C, respectively (Figure 1A). The area under the curve clearly showed a significant difference in thermogenesis between groups (Figure 1B).

### Cell Proliferation Following Irradiation

**FIGURE 2:**
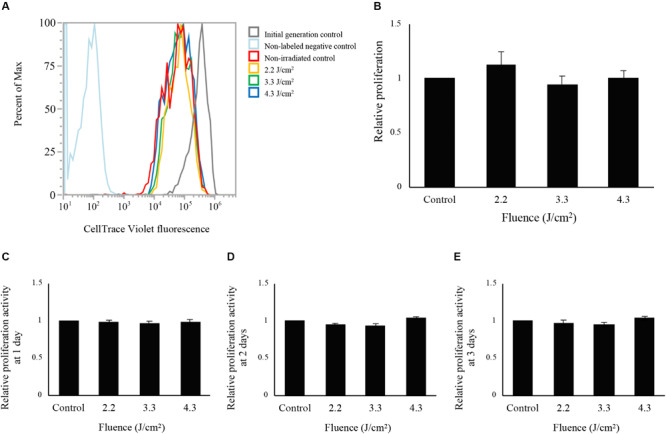
Evaluation of cell proliferation after low-level Er:YAG laser irradiation in osteoblast-like cells. **(A)** Fluorescence intensity distributions of cells labeled with CellTrace Violet at 24 h after Er:YAG laser irradiation. Gray line, initial-generation control; light blue, non-labeled negative control; red line, non-irradiated control; yellow line, cells irradiated at 2.2 J/cm^2^; green line, cells irradiated at 3.3 J/cm^2^; blue line, cells irradiated at 4.3 J/cm^2^. **(B)** Relative proliferation of cells irradiated by low-level Er:YAG laser at 1 day after irradiation compared to non-irradiated control cells by flow cytometer (means ± SE, *n* = 6) (steel method for comparison with non-irradiated cells). Relative proliferation of cells irradiated by low-level Er:YAG laser at **(C)** 1 day, **(D)** 2 days, and **(E)** 3 days after irradiation compared to non-irradiated control cells by CCK-8 (means ± SE, *n* = 6) (steel method for comparison with non-irradiated cells).

The fluorescence intensity distributions of cells labeled by CellTrace Violet at 24 h following irradiation are shown in Figure 2A. Because CellTrace Violet dye molecules are sequentially halved by cell division, cell proliferation measurements can be derived from the sequential reduction of its fluorescence (Kumar et al., 2013). Cells irradiated at 2.2, 3.3, and 4.3 J/cm^2^ and non-irradiated control cells showed a shift of the histogram peak toward the left relative to initial-generation control cells, indicating proliferation during the 24-h period following irradiation. However, there was no significant difference in average fluorescence values between non-irradiated control and irradiated cells (Figure 2B).

Cell proliferation evaluated by CCK-8 are shown in Figures 2C–E. Laser irradiation at any fluence showed no significant differences in proliferation between non-irradiated and irradiated cells at 1, 2, and 3 days after laser irradiation (*n* = 6).

### Calcification in Osteoblast-Like Cells

**FIGURE 3:**
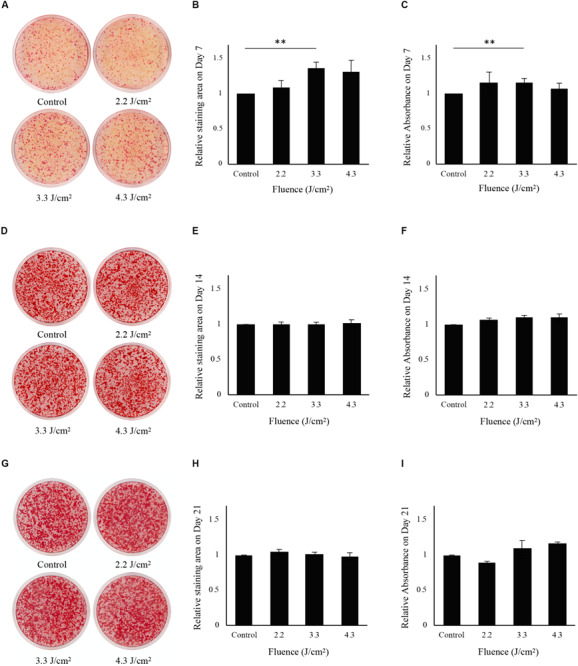
Evaluation of calcification in osteoblast-like cells after low-level Er:YAG laser irradiation. **(A)** Representative photographs of Alizarin red S staining on day 7 of non-irradiated cells and cells irradiated by Er:YAG laser at 2.2, 3.3, and 4.3 J/cm^2^. **(B)** Relative staining areas on day 7. **(C)** Relative absorbances in Alizarin red S staining after solubilization on day 7. **(D)** Representative photographs of Alizarin red S staining on day 14 of non-irradiated cells and cells irradiated by Er:YAG laser at 2.2, 3.3, and 4.3 J/cm^2^. **(E)** Relative staining areas on day 14. **(F)** Relative absorbances in Alizarin red S staining after solubilization on day 14. **(G)** Representative photographs of Alizarin red S staining on day 21 of non-irradiated cells and cells irradiated by Er:YAG laser at 2.2, 3.3, and 4.3 J/cm^2^. **(H)** Relative staining areas on day 21. **(I)** Relative absorbances in Alizarin red S staining after solubilization on day 21 (means ± SE, *n* = 6). **P < 0.01 (steel method for comparison with non-irradiated cells).

Osteoblast-like cells showed calcification after osteoinduction (Supplementary Figures S1A–C). Calcification data from 7 days after Er:YAG laser irradiation at 2.2, 3.3, and 4.3 J/cm^2^ is shown in Figure 3A. Calcification was significantly increased in cells irradiated at 3.3 J/cm^2^ compared to non-irradiated control cells. However, there was no significant difference in Alizarin red S staining area between cells irradiated at 2.2 or 4.3 J/cm^2^ and non-irradiated control cells (Figure 3B). The relative absorbances of solutions extracted from stained cells irradiated at 3.3 J/cm^2^ were significantly higher than those of non-irradiated control cells (Figure 3C).

Both non-irradiated and irradiated cells showed considerable mineral deposits at 14 and 21 days after irradiation (Figures 3D,G). The relative areas stained by Alizarin red S for all fluences showed no significant differences relative to those of non-irradiated control cells on days 14 and 21 (Figures 3E,H). There were no significant differences in relative absorbances of solutions extracted from stained cells between irradiated and control cells at 14 and 21 days after irradiation (Figures 3F,I).

### mRNA Expression in Osteoblast-Like Cells During Differentiation to Osteocyte

**FIGURE 4:**
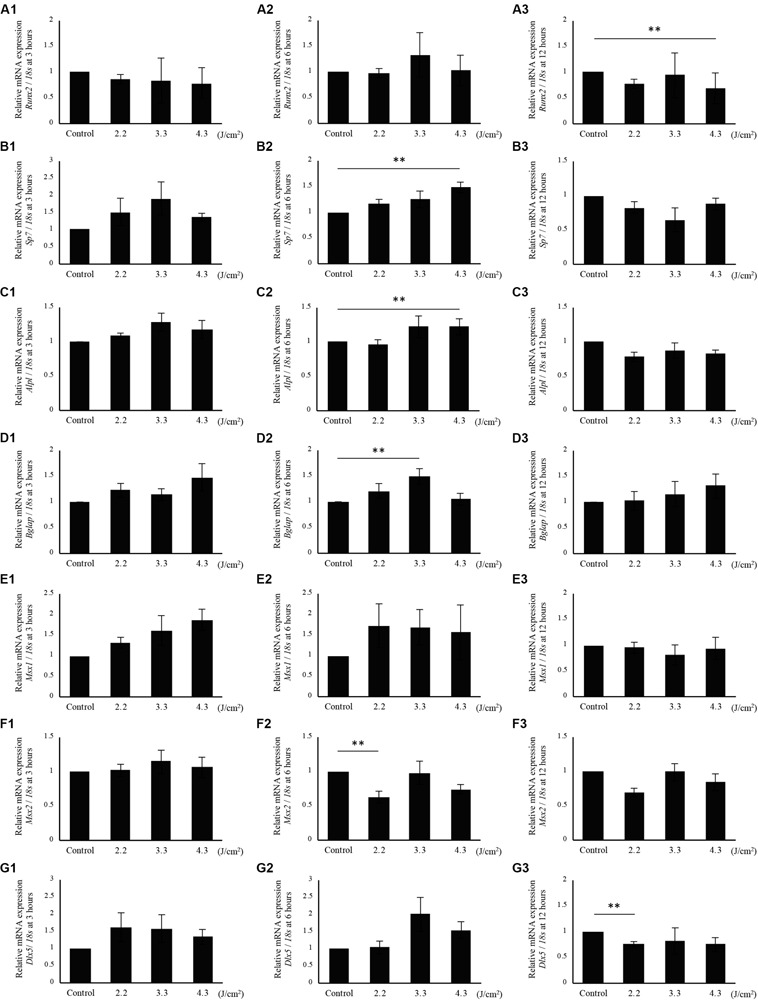
qPCR analysis of mRNA expression in osteoblast-like cells irradiated by low-level Er:YAG laser. *Runx2* expression at **(A1)** 3 h, **(A2)** 6 h, and **(A3)** 12 h after irradiation. *Sp7* expression at **(B1)** 3 h, **(B2)** 6 h, and **(B3)** 12 h after irradiation. *Alpl* expression at **(C1)** 3 h, **(C2)** 6 h, and **(C3)** 12 h after irradiation. *Bglap* expression at **(D1)** 3 h, **(D2)** 6 h, and **(D3)** 12 h after irradiation. *Msx1* expression at **(E1)** 3 h, **(E2)** 6 h, and **(E3)** 12 h after irradiation. *Msx2* expression at **(F1)** 3 h, **(F2)** 6 h, and **(F3)** 12 h after irradiation. *Dlx5* expression at **(G1)** 3 h, **(G2)** 6 h, and **(G3)** 12 h after irradiation (means ± SE, *n* = 5–6). **P < 0.01 (steel method for comparison with non-irradiated cells).

Since calcification of osteoblast-like cells was significantly increased after 3.3 J/cm^2^ Er:YAG laser irradiation, we evaluated *Runx2*, *Sp7*, *Alpl*, *Bglap*, *Msx1*, *Msx2*, and *Dlx5* mRNA expression (Figures 4A1–G3). *Bglap* expression was dramatically increased after osteoinduction (Supplementary Figure S1D). Irradiation produced no significant changes at 3 h in *Runx2* expression (Figure 4A1). *Runx2* expression tended to increase in cells irradiated at 3.3 J/cm^2^ after 6 h, but the difference was not significant (Figure 4A2). *Runx2* expression in cells irradiated at 4.3 J/cm^2^ significantly decreased at 12 h after irradiation (Figure 4A3). Meanwhile, the relative expression of *Sp7* in cells irradiated at 2.2 and 3.3 J/cm^2^ tended to increase at 3 and 6 h, but the increase did not reach statistical significance (Figures 4B1,B2). At 6 h after laser irradiation, *Sp7* expression in cells irradiated at 4.3 J/cm^2^ was significantly increased over control levels (Figure 4B2). No significant differences in *Sp7* expression were observed at 12 h (Figure 4B3). *Alpl* expression at 6 h after irradiation at 4.3 J/cm^2^ was significantly increased over control levels. Relative expression of *Alpl* in cells irradiated at 3.3 J/cm^2^ tended to increase at 3 and 6 h after laser irradiation, but differences were not significant (Figures 4C1,C2). Also, no significant difference was observed in *Alpl* expression between cells irradiated at 2.2 J/cm^2^ and control cells (Figures 4C1–C3). *Bglap* expression was significantly increased in cells irradiated at 3.3 J/cm^2^ after 6 h (Figure 4D2). When cells were irradiated at 4.3 J/cm^2^, the expression of *Bglap* at 3 and 12 h tended to increase, although the increase was not statistically significant. There was no significant difference in *Bglap* expression between cells irradiated at 2.2 J/cm^2^ and control cells (Figures 4D1–D3). No significant changes were observed in *Msx1* expression at 3, 6, and 12 h after laser irradiation at any fluences (Figures 4E1–E3). However, Er:YAG laser-irradiated cells tended to be increased *Msx1* expression at 3 and 6 h (Figures 4E1,E2). Cells irradiated at 2.2 J/cm^2^ had significantly decreased *Msx2* expression at 6 h, although no significant differences were observed at 3 and 12 h (Figures 4F1–F3). *Dlx5* expression in laser-irradiated cells at 3.3 and 4.3 J/cm^2^ tended to increase at 3 and 6 h, but there was no significant difference (Figures 4G1,G2). In particular, cells irradiated at 3.3 J/cm^2^ showed a strong increase at 6 h (Figure 4G2). Relative expression of *Dlx5* in cells irradiated at 2.2 J/cm^2^ was significantly decreased at 12 h after irradiation (Figure 4G3).

**FIGURE 5:**
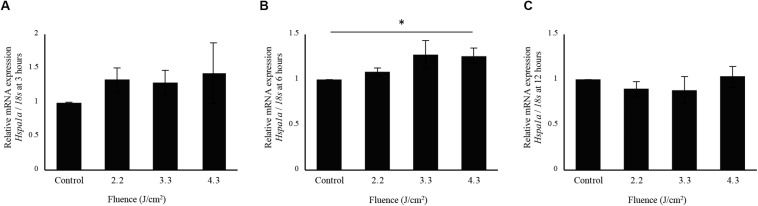
qPCR analysis of *Hspa1a* expression in osteoblast-like cells irradiated by-low-level Er:YAG laser. *Hspa1a* expression at **(A)** 3 h, **(B)** 6 h, and **(C)** 12 h after irradiation (means ± SE, *n* = 5–6). *P < 0.05 (steel method for comparison with non-irradiated cells).

Interestingly, cells irradiated at 4.3 J/cm^2^ showed significantly higher *Hspa1a* expression compared to control cells at 6 h after irradiation. There was no significant increase in *Hspa1a* expression after irradiation at 2.2 and 3.3 J/cm^2^ (Figures 5A–C).

**FIGURE 6:**
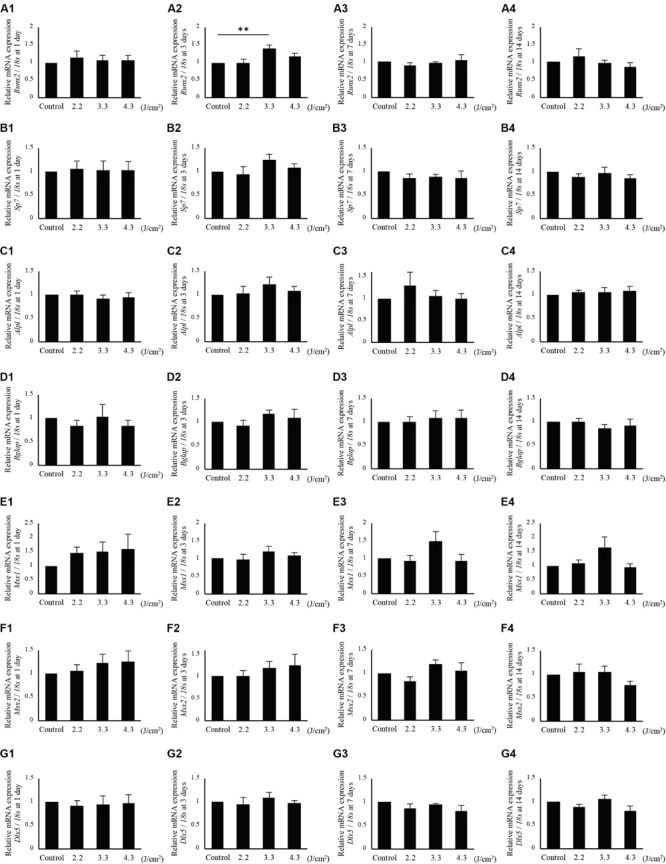
qPCR analysis of mRNA expression in osteoblast-like cells irradiated by low-level Er:YAG laser. *Runx2* expression at **(A1)** 1 day, **(A2)** 3 days, **(A3)** 7 days, and **(A4)** 14 days after irradiation. *Sp7* expression at **(B1)** 1 day, **(B2)** 3 days, **(B3)** 7 days, and **(B4)** 14 days after irradiation. *Alpl* expression at **(C1)** 1 day, **(C2)** 3 days, **(C3)** 7 days, and **(C4)** 14 days after irradiation. *Bglap* expression at **(D1)** 1 day, **(D2)** 3 days, **(D3)** 7 days, and **(D4)** 14 days after irradiation. *Msx1* expression at **(E1)** 1 day, **(E2)** 3 days, **(E3)** 7 days, and **(E4)** 14 days after irradiation. *Msx2* expression at **(F1)** 1 day, **(F2)** 3 days, **(F3)** 7 days, and **(F4)** 14 days after irradiation. *Dlx5* expression at **(G1)** 1 day, **(G2)** 3 days, **(G3)** 7 days, and **(G4)** 14 days after irradiation (means ± SE, *n* = 6). **P < 0.01 (steel method for comparison with non-irradiated cells).

The relative expression levels of *Runx2*, *Sp7, Alpl*, *Bglap*, *Msx1*, *Msx2*, and *Dlx5* at 1, 3, 7, and 14 days after laser-irradiation are shown in Figures 6A1–G4. *Runx2* expression in cells irradiated at 3.3 J/cm^2^ was significantly increased at 3 days after irradiation, although there were no significant differences between irradiated and non-irradiated control cells at 1, 7, and 14 days after irradiation (Figures 6A1–A4). In addition, no significant differences were observed in *Sp7*, *Alpl*, *Bglap*, *Msx1*, *Msx2*, and *Dlx5* expression between irradiated and non-irradiated control cells after 1, 3, 7, and 14 days (Figures 6B1–G4). However, *Sp7* expression at 3 days (Figure 6B2) and *Msx1* expression at 7 and 14 days (Figures 6E3,E4) tended to be increased in cells irradiated at 3.3 J/cm^2^.

### Microarray Analysis

**FIGURE 7:**
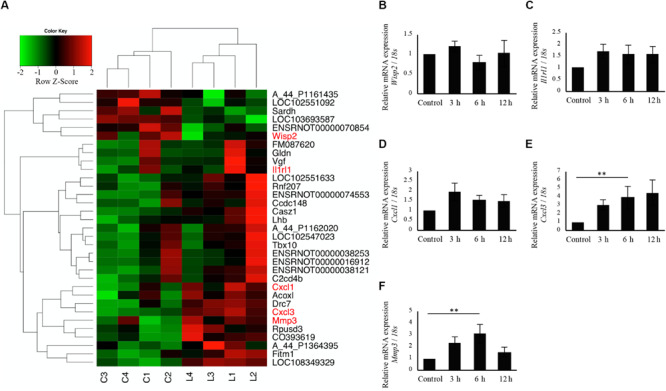
Comprehensive evaluation of gene expression between non-irradiated cells and irradiated cells 6 h after Er:YAG laser irradiation at 3.3 J/cm^2^ using microarray expression profiling. **(A)** Heatmaps of genes showing *P* < 0.01 (*n* = 4) (green, low; red, high) (C, non-irradiated control cells, L, laser irradiated cells). qPCR analysis of mRNA expressions in cells 3, 6, and 12 h after Er:YAG laser irradiation at 3.3 J/cm^2^; **(B)** *Wisp2*, **(C)** *Il1rl1*, **(D)** *Cxcl1*, **(E)** *Cxcl3*, and **(F)** *Mmp3* (means ± SE, *n* = 5–6). **P < 0.01 (steel method for comparison with non-irradiated cells).

Since calcification and *Bglap* expression at 6 h following irradiation were significantly increased in cells irradiated at 3.3 J/cm^2^, microarray analysis was performed to obtain a comprehensive overview of the gene expression profile (control, *n* = 4; cells at 6 h after 3.3 J/cm^2^ irradiation, *n* = 4). All microarray data are shown in Supplementary Table S2. No genes showed |fold change| >2 and *q* < 0.1. However, 33 genes showed changes for which *P* < 0.01 (Figure 7A). We subsequently focused on genes related to inflammation and *Wisp2*, validating the microarray data by qPCR. *Wisp2* expression showed no significant difference, however, it tended to decrease in irradiated cells at 6 h after irradiation at 3.3 J/cm^2^ (Figure 7B). The expression of *Il1rl1* and *Cxcl1* on cells irradiated at 3.3 J/cm^2^ did not differ significantly at any time point after irradiation (Figures 7C,D). There were significant increases in *Cxcl3* and *Mmp3* expression at 6 h after irradiation (Figures 7E,F). Microarray data from this study are available in the Gene Expression Omnibus database^2^ as GSE146098.

### Gene Set Enrichment Analysis

**FIGURE 8:**
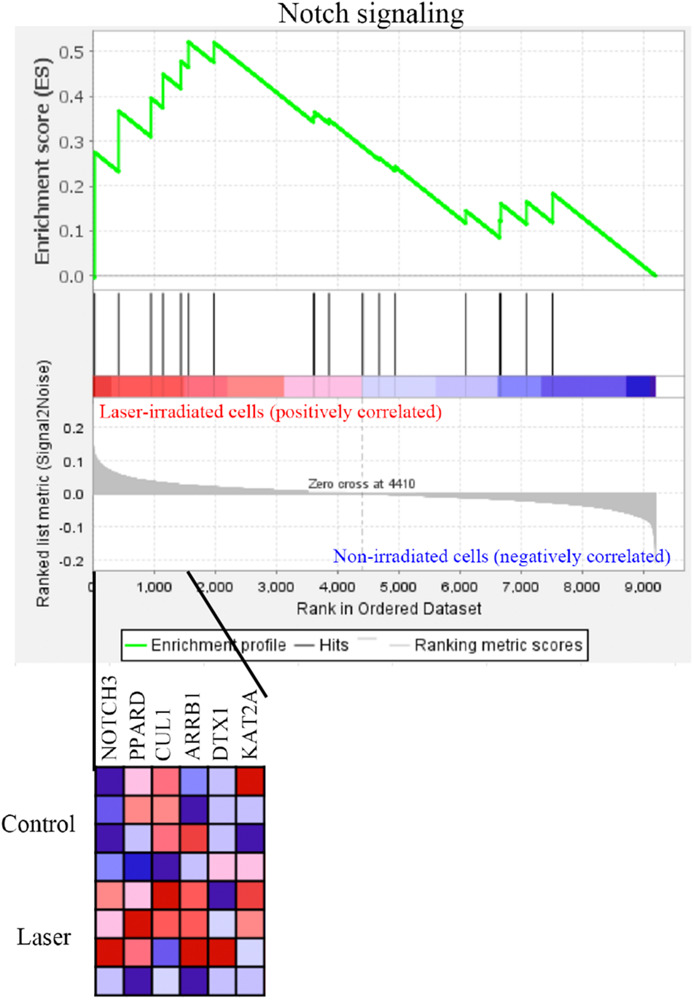
GSEA with hallmark gene sets enriched in Er:YAG laser-irradiated cells at 3.3 J/cm^2^ after 6 h (*n* = 4). Gene set related to Notch signaling. A heatmap is provided to illustrate the gene expression levels for each gene in the core enrichment subset (blue, low; red, high).

GSEA was performed using hallmark gene sets to evaluate the gene expression changes caused by 3.3 J/cm^2^ laser irradiation at 6 h. The gene sets enriched in irradiated cells were identified with FDR-q < 0.25 (Table 1). The details for the Notch Signaling gene set [normalized enrichment score (NES) = 1.43, *q* = 0.138] are shown in Figure 8.

**TABLE 1:** Gene set enrichment analysis at 6 h after Er:YAG irradiation.

**Gene Set**	**Size**	**NES**	**Normal *P*-value**	**FDR-q value**
IL2 STAT5 signaling	125	1.66	< 0.001	0.056
IL6 JAK STAT3 signaling	49	1.60	0.007	0.046
Notch signaling	18	1.43	0.083	0.138

## Discussion

To evaluate the effect of low-level Er:YAG laser irradiation itself on cells, fluences from 2.2 to 4.3 J/cm^2^ were used in this study. These low fluences limited temperature increases to a maximum of 4.6°C at the highest fluence, and 3.4°C at 3.3 J/cm^2^. In addition, mRNA expression of the heat shock protein-related gene *Hspa1a* showed no statistically significant increase at 6 h after laser irradiation at 3.3 J/cm^2^. It was considered that there was little thermal effect by irradiation at 3.3 J/cm^2^ of Er:YAG laser in this study.

Based on a previous report that the doubling time of rat calvarial osteoblasts was approximately 2–3 days at passage 2–5 (Liu et al., 2019), we evaluated the proliferation of rat calvarial osteoblast-like cells on 1, 2, and 3 days after irradiation. None of the fluences significantly increased proliferation. We also evaluated proliferation on 1 day after irradiation by flow cytometry of cells labeled with CellTrace Violet. This novel technique directly measures cell division, and showed that Er:YAG laser irradiation itself neither induced cell toxicity nor enhanced cell proliferation. However, the cell density and the culturing period were limited to prevent cells from becoming confluent, and thus flow cytometry was performed only on day 1 after irradiation. Further optimizations are needed to apply this innovative method in longer-term culture conditions. A previous study reported that Er:YAG laser irradiation significantly increased proliferation of the osteoblastic cell line MC3T3-E1 on days 1 and 3 after irradiation at fluences similar to those used in our study (2.1, 3.6, 4.3 J/cm^2^) (Aleksic et al., 2010). It is conceivable that the disparities in results are caused by differences in irradiation conditions (pulse energy and time) and/or cells used. In addition, although we previously reported that Er:YAG irradiation at 6.3 J/cm^2^ promoted cell proliferation of human gingival fibroblasts, surface temperatures increased to 40.9°C with upregulating genes of heat shock protein family proteins (Kong et al., 2018). The difference in temperature may also affect proliferation.

Calcification of osteoblasts was promoted by laser irradiation, in agreement with some previous studies using various types of lasers (Ozawa et al., 1998; Ueda and Shimizu, 2003; Mikami et al., 2018). Mikami et al. (2018) reported enhanced calcification of MC3T3-E1 cells (analyzed by Alizarin red S staining) via TRPV1 by 405-nm ultrahigh-frequency and ultrashort-pulse blue laser irradiation at 5.6 J/cm^2^ and 80 MHz. Regarding primary osteoblast-like cells isolated from rat calvariae, Ozawa et al. (1998) observed that Ga-Al-As diode laser irradiation at 3.82 J/cm^2^ on days 1–12, respectively, increased calcification. Ueda and Shimizu (2003) also reported enhanced bone nodule formation by primary osteoblast-like cells at day 21 by Ga-Al-As laser irradiation at 3.84 J/cm^2^. Irradiation with a Ga-Al-As laser on day 3 at 3.82 J/cm^2^ also increased bone nodule formation by osteoblast-like cells at day 24 post-irradiation (Shimizu et al., 2007). However, the effects of Er:YAG laser irradiation on calcification of osteoblasts have not been reported previously. In the present study, we clearly showed that Er:YAG laser irradiation at 3.3 J/cm^2^ significantly enhanced extracellular calcification of osteoblasts in vitro. In addition, the expression of *Bglap*, a marker of osteoblast terminal differentiation (Ikeda et al., 1992), increased significantly in cells 6 h after irradiation at 3.3 J/cm^2^. This result is associated with the promoted calcification of osteoblasts by Er:YAG laser irradiation. We also reported that *Bglap* expression was significantly increased in the granulation tissues of Er:YAG laser-ablated bone defects compared to that of bur-drilled bone defects at 1 week after treatment in rat (Ohsugi et al., 2019). A previous study reported that mineralization of rat calvarial osteoblasts was initiated when cultured in osteogenic induction medium for 3 days, which was earlier than that of mouse osteoblasts on day 7 (Liu et al., 2019). In this study, we also observed the calcification of osteoblast-like cells only at 7 days after osteogenic induction.

The expression of *Sp7*, which is normally required for *Bglap* activation and bone formation (Nakashima et al., 2002), tended to increase in cells 3 h after irradiation at 3.3 J/cm^2^. At 6 h, cells irradiated at 4.3 J/cm^2^ significantly enhanced expression of *Sp7*. Laser irradiation increased the expression of *Sp7* at 3 and 6 h, which possibly resulted in significantly enhanced expression of *Bglap* at 6 h in cells irradiated at 3.3 J/cm^2^ and a trend toward increased *Bglap* expression in irradiated cells at 12 h. Furthermore, focusing on the *Sp7* and its regulation of target genes *Alpl* and *Bglap*, the expression pattern of *Alpl* at 3 h was similar to that of *Sp7* at 3 h. In addition, the expression pattern of *Bglap* at 6 h resembled that of *Sp7* at 3 h. These results suggest that *Bglap* expression may be induced by the increase of *Sp7* expression, in accordance with a previous report (Nakashima et al., 2002). *Dlx5* (Distal-less homeobox 5) is specifically expressed in osteogenic cells and stimulates osteoblast differentiation, activating osteocalcin promoters and other bone marker genes (Ryoo et al., 1997; Newberry et al., 1998; Miyama et al., 1999; Lee et al., 2003; Kim et al., 2004). The relative expression of *Dlx5* in cells irradiated at 3.3 J/cm^2^ tended to be increased at 6 h after laser irradiation, which corresponded with a significant increase of *Bglap* expression in cells irradiated at 3.3 J/cm^2^ at 6 h. These results suggest that Er:YAG laser irradiation may promote *Bglap* expression by enhancing the expression of *Sp7* and *Dlx5* in bone tissue. While the expression of *Sp7* in cells irradiated at 4.3 J/cm^2^ exhibited a significant increase at 6 h after irradiation, *Runx2* expression in cells irradiated at 4.3 J/cm^2^ was significantly decreased at 12 h. Previous studies reported mutual regulation between *Runx2* and genes in its signaling pathways, including *Sp7* (Kawane et al., 2014; Komori, 2020). In this study, irradiation at 4.3 J/cm^2^ did not enhance calcification significantly in osteoblast-like cells. This result suggests that Er:YAG laser irradiation at 4.3 J/cm^2^ is too much energy to induce cell calcification. *Msx1* (Msh homeobox 1) regulates osteoblast differentiation and promotes osteogenic proliferation (Nassif et al., 2014; Goto et al., 2016). The relative expression of *Msx1* showed a tendency to increase in laser-irradiated cells at 3 and 6 h after laser irradiation. Msx2 (Msh homeobox 2) is reported to promote the proliferation of osteoprogenitors in the early stage of cell differentiation, but prevent terminal osteoblast differentiation as a repressor (Dodig et al., 1999; Shirakabe et al., 2001; Kim et al., 2004). In this present study, the expression of *Msx2* was not significantly increased by Er:YAG laser irradiation, suggesting that irradiation did not prevent osteogenic differentiation.

While a short-term mRNA expression profile demonstrated that laser irradiation-induced beneficial effects for osteoblast differentiation, gene expression did not show dramatic changes after Er:YAG laser irradiation in the long-term. Irradiation might have affected the early stages of osteoblast differentiation. Alternatively, since irradiation was only performed once in this study, multiple irradiations might induce more beneficial effects for osteoblast differentiation.

Microarray analysis showed that irradiation at 3.3 J/cm^2^ caused downregulation of *Wisp2* at 6 h, although we could not validate significant downregulation of *Wisp2* by qPCR. *Wisp2*, also known as *Ccn5*, plays an important role in differentiation and mineralizing osteoblasts (Kawaki et al., 2011). A previous report clarified that *Wisp2*/*Ccn5* knockdown increased *Bglap* expression (Kawaki et al., 2011), suggesting that Er:YAG laser irradiation on osteoblast-like cells enhanced the expression of *Bglap* by inhibiting *Wisp2*.

Interestingly, the inflammation-related genes *Cxcl3* and *Mmp3* were significantly upregulated 6 h after Er:YAG laser irradiation at 3.3 J/cm^2^. *Cxcl3* (C-X-C Motif Chemokine Ligand 3) is transcriptionally activated by IL-1 via the NF-κB signaling pathway (Anisowicz et al., 1991), and Mmp3 (Matrix metalloproteinase-3), also known as Stromelysin-1, is induced by IL-1β and TNFα (Meller et al., 2000). Our results showed that Er:YAG laser irradiation at 3.3 J/cm^2^ caused inflammation. However, considering that there was no dramatic increase in temperature, the inflammation possibly resulted from non-thermal effects, such as photo-mechanical stimulation. A previous study revealed that laser irradiation has the potential to induce mechanical stress (Yokose, 2013), which has been reported to affect bone modeling positively (Rosa et al., 2015; Shimohira et al., in press).

Gene set enrichment analysis revealed enrichment of the Notch signaling by irradiation. This pathway is highly evolutionarily conserved and plays a critical role in a variety of cellular functions. It also has been reported to promote osteogenic differentiation of osteoblasts in synergy with BMP (Lin and Hankenson, 2011). Activation of Notch signaling in MC3T3-E1 cells by adenoviral overexpression of the Notch1 cytoplasmic domain Notch-IC (NIC) caused a significant increase in calcified nodule formation (Tezuka et al., 2002). A previous study demonstrated that overexpressing Notch ligands and treatment with BMP-2 in MC3T3-E1 resulted in enhanced ALP activity and in vitro ectopic bone formation (Nobta et al., 2005). Thus, it is possible that calcification of irradiated osteoblast-like cells was enhanced via the Notch signaling.

A limitation of this study was its use of primary osteoblast-like cells from rat calvariae rather than osteoblasts. Several studies have isolated osteoblast-like cells from mouse or rat calvariae, with cells from fractions 2–5 of enzymic digestion used as osteoblast-like cells (Ozawa et al., 1998; Ogata et al., 2000; Ueda and Shimizu, 2003; Fukuhara et al., 2006; Shimizu et al., 2007; Nakashima et al., 2011). Gu et al. (2006) identified osteoblast-like cells based on morphology and staining for ALP. The cells from fraction 3 and 4 of enzymic digestion included 50.3% of osteoblast-like cells with ALP-positive and cuboidal shape; however, the cells from fraction 5 included only 29.1% osteoblast-like cells (Gu et al., 2006). Therefore, we used “osteoblast-like cells” isolated from rat calvaria from fractions 2–4 by serial enzymatic digestion, similar to previous studies.

## Conclusion

In conclusion, this is the first study to show enhanced calcification after Er:YAG laser irradiation and to comprehensively evaluate gene expression in osteoblast-like cells. Although the stimulatory effect of low-level laser irradiation was only short-term for osteoblastic differentiation, irradiation especially at 3.3 J/cm^2^ enhanced calcification of primary osteoblast-like cells without major thermal effects. The promotion of mineralization of osteoblast-like cells may be associated with enhanced *Bglap* expression as well as enriched Notch signaling. Our results showed Er:YAG laser irradiation at 3.3 J/cm^2^ might represent an optimum fluence for promotion of bone formation.

## Data Availability Statement

The datasets generated for this study can be found in the Gene Expression Omnibus database/GSE146098.

## Ethics Statement

The animal study was reviewed and approved by the Animal Care Committee of the Experimental Animal Center at Tokyo Medical and Dental University.

## Author Contributions

HN performed most of the experiments and wrote the 1st draft of the manuscript. YO, KW, MH, TS, YT, SM, TH, HK, SY, TI, and AA assisted in some studies and reviewed the manuscript. MH and TH provided expertise on microarray analysis. SK and AA supervised all the studies and the writing of the manuscript.

## Conflict of Interest

The authors declare that the research was conducted in the absence of any commercial or financial relationships that could be construed as a potential conflict of interest.

## Footnotes

1 http://software.broadinstitute.org/gsea/index.jsp

2 www.ncbi.nlm.nih.gov/geo
