# Supplementary figures and images for: Effects of Low-Level Er:YAG Laser Irradiation on Proliferation and Calcification of Primary Osteoblast-Like Cells Isolated From Rat Calvaria
Source: Front Cell Dev Biol. 2020 Jun 23;8:459. doi: 10.3389/fcell.2020.00459 (PMC7324552; doi:10.3389/fcell.2020.00459)

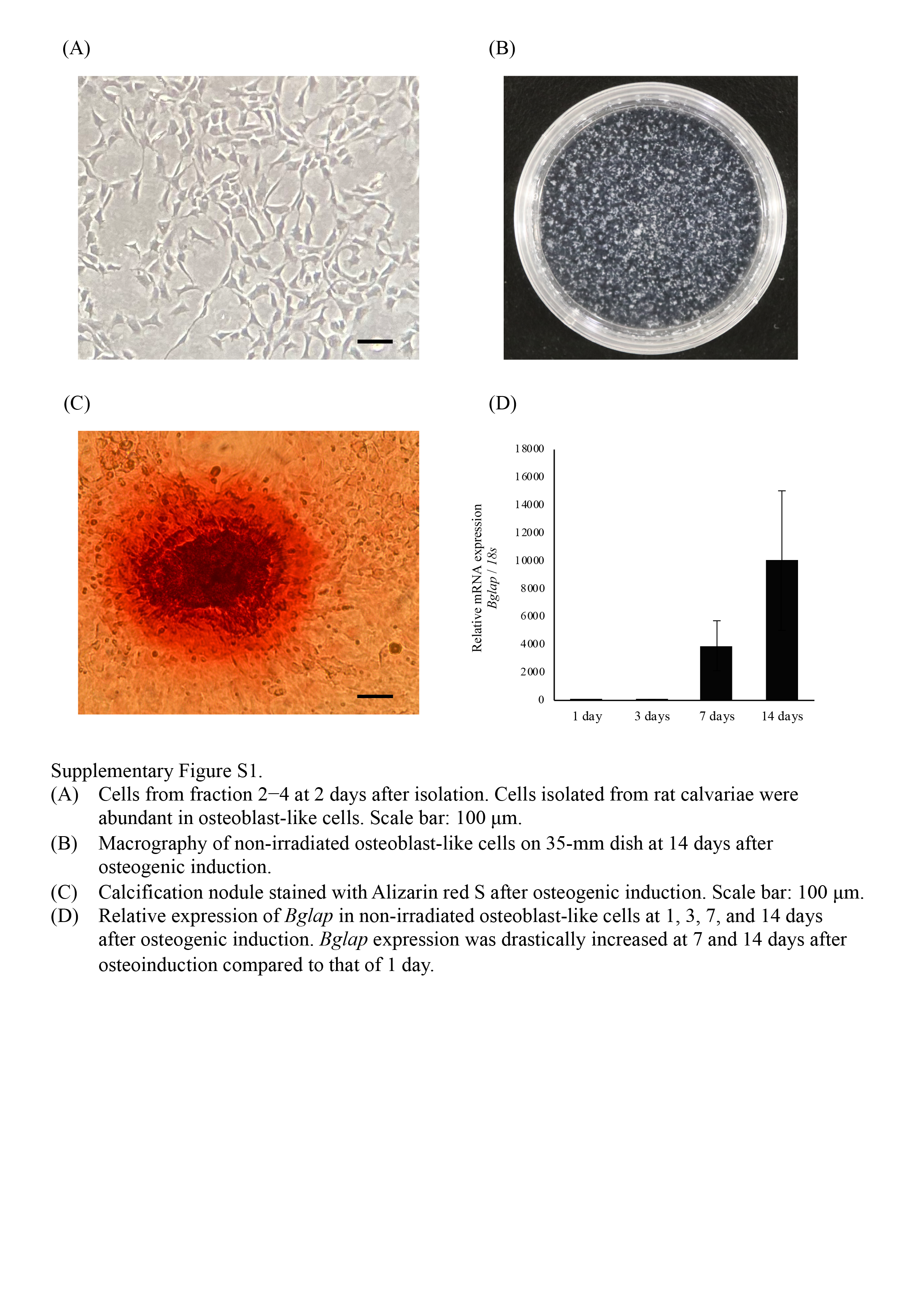

Supplement: Supplementary file 3 [file Image_1.tif]
